# *In Silico* Studies of Quinoxaline-2-Carboxamide 1,4-di-*N*-Oxide Derivatives as Antimycobacterial Agents

**DOI:** 10.3390/molecules19022247

**Published:** 2014-02-21

**Authors:** Awwad A. Radwan, Wael M. Abdel-Mageed

**Affiliations:** 1 Department of Pharmaceutical Organic Chemistry, Faculty of Pharmacy, Assiut University, Assiut 71526, Egypt; 2 Department of Pharmaceutics, College of Pharmacy, King Saud University, Riyadh 11451, Saudi Arabia; 3 Pharmacognosy Department, College of Pharmacy, King Saud University, Riyadh 11451, P. O. Box 2457, Saudi Arabia; E-Mail: wabdelmageed@ksu.edu.sa; 4 Pharmacognosy Department, Faculty of Pharmacy, Assiut University, Assiut 71526, Egypt

**Keywords:** pharmacophore, homology modeling, molecular docking, Dock6, antimycobacterial activity

## Abstract

Molecular modelling studies were performed on some previously reported novel quinoxaline-2-carboxamide 1,4-di-*N*-oxide derivatives (series 1–9). Using the LigandScout program, a pharmacophore model was developed to further optimize the antimycobacterial activity of this series of compounds. Using the Dock6 program, docking studies were performed in order to investigate the mode of binding of these compounds. The molecular modeling study allowed us to confirm the preferential binding mode of these quinoxaline-2-carboxamide 1,4-di-*N*-oxide derivatives inside the active site. The obtained binding mode was as same as that of the novobiocin X-ray structure.

## 1. Introduction

Tuberculosis (TB) is the most prevalent infectious bacterial disease caused by *Mycobacterium tuberculosis* (*mtb*). In 2009, the World Health Organization (WHO), reported an estimated 9.27 million cases of *mtb* in 2007. A remarked increase was noticed from the 9.24 million cases in 2006, the 8.3 million cases in 2000 and the 6.6 million cases in 1990. While the total number of incident cases of *mtb* ius increasing, the number of *mtb* infected cases *per capita* is slowly decreasing. A leading killer, tuberculosis is an intracellular infection responsible for some 3 million deaths annually, with a person lost to *mtb* every 15 s [1]. Readily spreading from person to person, and showing bad resistance to isoniazide and rifamicin, multidrug-resistant strains of *M. tuberculosis* (MDRTB) will necessarily make the future control of TB more difficult. This dilemma was worsened by the emergence of XDR-TB, that is unresponsive not only to isoniazid and rifampicin, the first line drugs, but also to a fluoroquinolone and to at least one of the second-line drugs (amikacin, capreomycin or kanamycin) [2]. In addition to these drug-resistance characteristics of TB, the recent influx of immigrants from countries endemic for the disease and co-infection with human immunodeficiency virus (HIV) [3,4] highlight the urgent need for new drugs to extend the range of effective TB treatment options. Quinoxaline derivatives are an attractive class of target compounds for new drug development because of their potentially versatile biological activities, including antiviral, anticancer, antibacterial and antiprotozoal properties [5,6,7,8,9]. As antituberculous agents, a wide range of quinoxaline-1,4-di-*N*-oxide derivatives with variable substituents at different positions were reported [10,11,12,13,14,15,16,17,18].

As anti-*T. cruzi* agents, QSAR studies of 3-arylquinoxaline-2-carbonitrile di-*N*-oxides were performed by establishing a link between IC_50_ values and their Moriguchi-octanol/water partition coefficients (MLOGPs) [19]. In the past decades, due to a lack of experimental biological data on quinoxaline derivatives as anti-mycobacterial agents, none of the studies have included quantitative structure activity results. With the addition of new data to the literature, research groups have undertaken further exploration of the biological profile exhibited by quinoxaline-2-carboxylate 1,4-di-*N*-oxide derivatives resorting to the QSAR formalism, establishing predictive models for biological properties [20].

In view of these findings, we are prompted to perform further studies, using molecular modeling software, in order to explore the structural requirements necessary for the anti-tuberculous activity of quinoxaline derivatives. Furthermore, *in silico* interactions of the quinoxaline derivatives under study within the *mtb*-DNA gyrase active site could provide valuable information for their possible mode of action.

## 2. Results and Discussion

### 2.1. Pharmacophore Modeling

**Figure 1:**
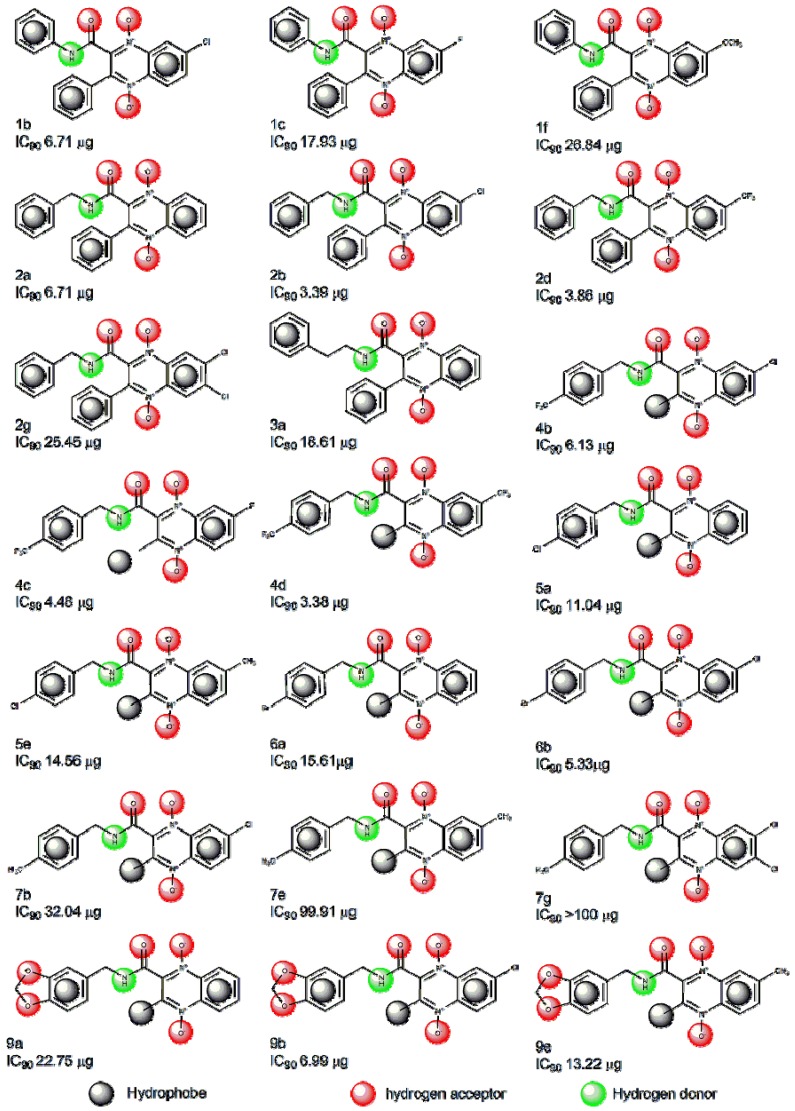
Structures of the quinoxaline-2-carboxamide 1,4-di-*N*-oxide compounds used in the LigandScout training set [16].

**Figure 2:**
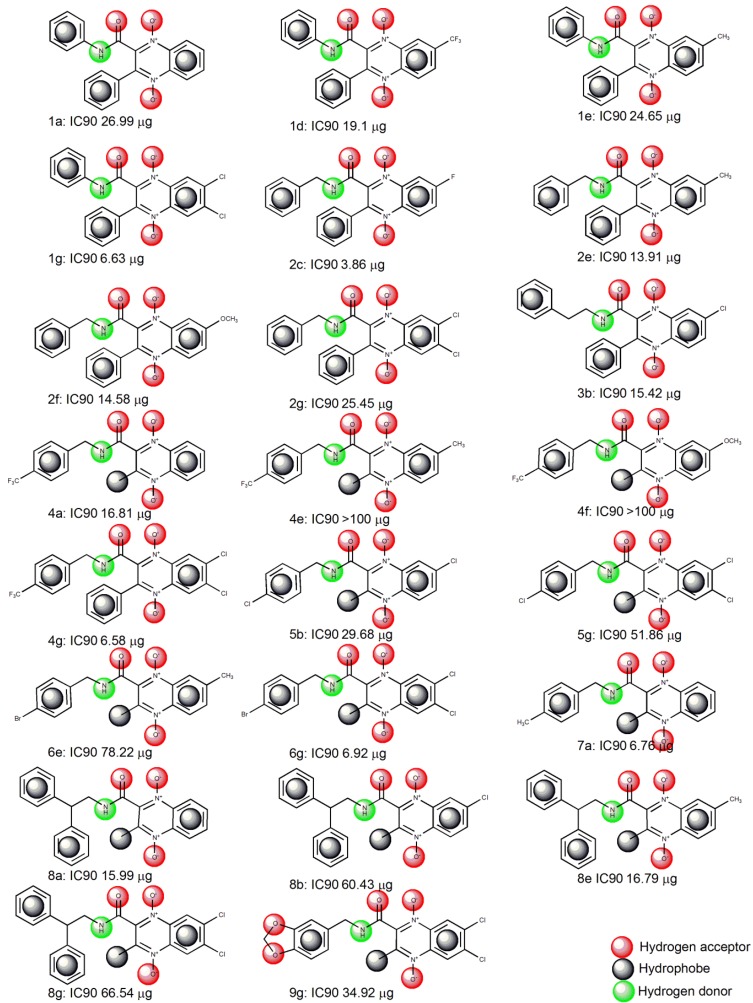
Structures of the quinoxaline-2-carboxamide 1,4-di-*N*-oxide compounds used in the LigandScout test set [16].

Elucidation of the binding approaches for the compounds under study is suggested based on finding the active structures. Figure 1 shows the structure of the training set compounds (**1b**,**c**,**f**, **2a**,**b**,**d**,**g**, **3a**, **4b**–**e**, **5a**,**e**, **6a**,**b**, **7b**,**e**,**g**, **9a**,**b**,**e**) and Figure 2 shows the structure of the test set compounds (**1a**,**d**,**e**,**g**, **2c**,**e**,**f**, **3b**, **4a**,**f**,**g**, **5b**,**g**, **6e**,**g**, **7a**, **8a**,**b**,**e**,**g**, **9g**). Based on the assumption that the active compounds bind in a similar fashion at the active site, the Ligandscout program [19,20,21] was employed to evaluate the common features essential for antiproliferative activity and the hypothetical geometries adopted by these ligands in their most active forms. Thus, these compounds were submitted for pharmacophore model generation based on the shared chemical features. Diverse conformations within a 20 kcal/mol energy range were generated and submitted to the alignment procedure.

**Figure 3:**
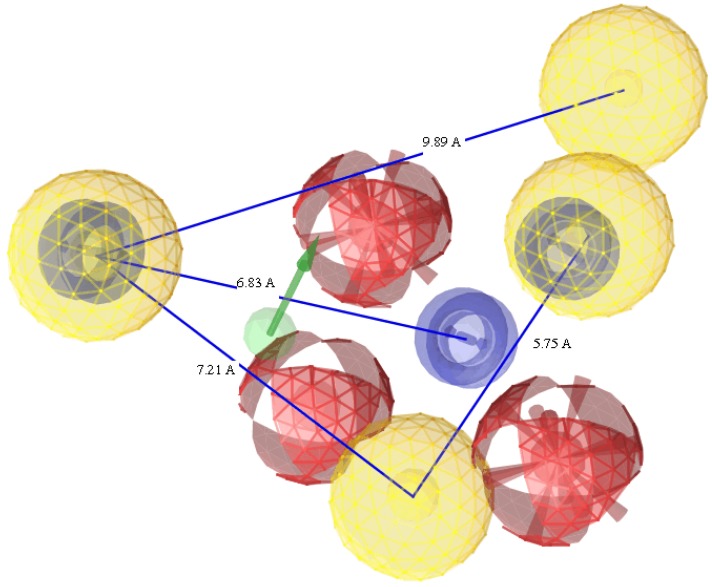
The top-ranked chemical feature-based pharmacophore model developed using the LigandScout program. The pharmacophore includes one aromatic rings (blue), four hydrophobes (yellow), three hydrogen acceptor feature (red) and one hydrogen donor (green). Distances are given in Angstrom.

The successful pharmacophore run resulted in generation of 10 hypotheses, as its highest rank score and mapping into all training set molecules, hypo1 was considered statistically as the best hypothesis and it was selected for further investigation and analysis. The top-ranked chemical feature-based pharmacophore model identified in this study is shown in Figure 3. This pharmacophore model contains nine chemical features: one aromatic ring (blue), four hydrophobes (yellow), three hydrogen acceptors (red) and one hydrogen donor (green). All the training set and test set compounds were mapped onto hypo1 with scoring the orientation of a mapped compound within the hypothesis features using a “fit value” score.

**Figure 4:**
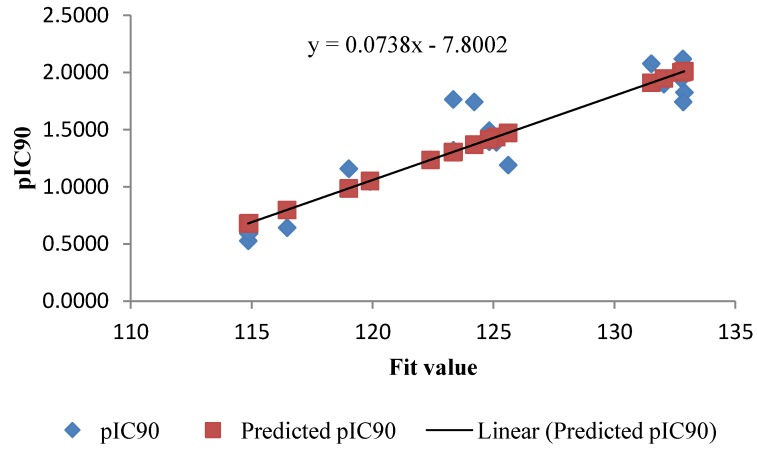
Fit plot of the predicted pIC90 of the training set compounds with its experimental pIC90.

**Figure 5:**
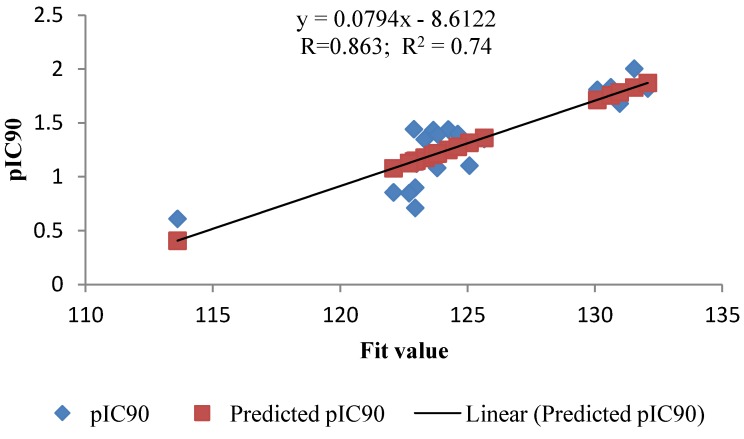
Fit plot of the predicted pIC90 of the test set compounds with its experimental pIC90.

As a quick and primary validation of hypo1, mapping of the compounds found to show a good agreement between the fit value and the biological activity (Table 1 and Table 2, Figure 4 and Figure 5). Initial investigation of the results shown in Table 1 and Table 2 revealed a moderate correlation between the fit value and the biological activity of each of the tested compounds. Of the training set, the highly active compounds (**1b**, **2b**, **4b**–**d**, **6b** and **9b**) showed a range of fit value of 131.46–132.89 whereas compounds with lower activity showed a lower fit value average of 125.6–114.85. This initial correlation encouraged us to generate a linear model based on “fit value” to predict the biological activity of the compounds under investigation. The generated model (Equation 1) showed good statistics and was used successfully to calculate the activity of the tested compounds (Figure 2):

pIC90 = 0.0738x − 7.8002
(1)

where number of compounds, x is the fit value and n = 22, st. error = 0.1895, R = 0.929 and R^²^ = 0.863. 

**Table 1:** Output for Hypo1 mapping and predictive model of training set compounds.

Compounds	IC90 μM	pIC90	Fit Value	*Predicted pIC90*	*Residuals*
**1b**	0.0172	1.7655	123.33	1.3065	0.4590
**1c**	0.0478	1.3205	123.34	1.3072	0.0133
**1f**	0.0694	1.1589	119.01	0.9875	0.1714
**2a**	0.0181	1.7427	124.2	1.3707	0.3720
**2b**	0.0084	2.0773	131.52	1.9113	0.1660
**2d**	0.2278	0.6425	116.46	0.7992	–0.1567
**2g**	0.0580	1.2368	122.39	1.2371	–0.0003
**3a**	0.0483	1.3157	123.32	1.3058	0.0099
**4b**	0.0149	1.8264	132.89	2.0125	–0.1861
**4c**	0.0113	1.9453	132.76	2.0029	–0.0576
**4d**	0.0076	2.1194	132.82	2.0073	0.1121
**4e**	0.2558	0.5922	114.85	0.6803	–0.0881
**5a**	0.0322	1.4923	124.82	1.4165	0.0758
**5e**	0.0408	1.3895	125.11	1.4379	–0.0484
**6a**	0.0402	1.3954	124.82	1.4165	–0.0211
**6b**	0.0126	1.8986	132.04	1.9497	–0.0511
**7b**	0.0897	1.0470	119.89	1.0525	–0.0055
**7e**	0.2965	0.5280	114.85	0.6803	–0.1523
**7g**	0.2551	0.5933	114.89	0.6832	–0.0899
**9a**	0.0644	1.1908	125.6	1.4741	–0.2833
**9b**	0.0181	1.7432	132.84	2.0088	–0.2656
**9e**	0.0360	1.4434	124.81	1.4158	0.0276

**Table 2:** Output for Hypo1 mapping and predictive model of test set compounds.

Compounds	IC90 μM	pIC90	Fit Value	*Predicted pIC90*	*Residuals*
**1a**	0.0756	1.1214	122.99	1.1506	–0.0292
**1d**	0.0449	1.3473	123.31	1.176	0.1713
**1e**	0.0664	1.1775	123.34	1.1784	–0.0009
**1g**	0.0156	1.8078	130.09	1.7142	0.0936
**2c**	0.0099	2.0033	131.54	1.8293	0.174
**2e**	0.0361	1.4421	122.89	1.1427	0.2994
**2f**	0.0364	1.4393	124.24	1.2498	0.1895
**3b**	0.0368	1.4341	123.65	1.203	0.2311
**4a**	0.0446	1.3507	125.65	1.3617	–0.011
**4f**	0.2457	0.6095	113.61	0.406	0.2035
**4g**	0.0148	1.8311	130.62	1.7563	0.0748
**5b**	0.0787	1.1038	125.07	1.3157	–0.2119
**5g**	0.1259	0.9	122.94	1.1466	–0.2466
**6e**	0.1946	0.7109	122.94	1.1466	–0.4357
**6g**	0.0151	1.8198	132.07	1.8714	–0.0516
**7a**	0.0209	1.6792	130.97	1.784	–0.1048
**8a**	0.0401	1.3971	124.61	1.2792	0.1179
**8b**	0.1396	0.8552	122.09	1.0792	–0.224
**8e**	0.0407	1.3908	123.83	1.2173	0.1735
**8g**	0.1422	0.8471	122.7	1.1276	–0.2805
**9g**	0.0827	1.0822	123.8	1.2149	–0.1327

**Figure 6:**
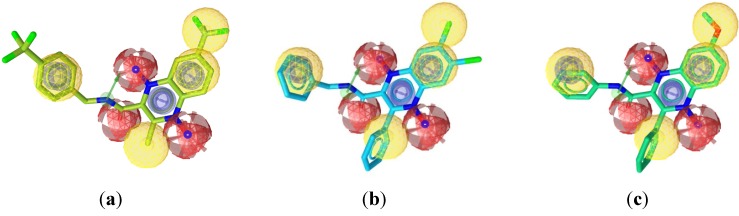
(**a**) Best aligned pose of compound **4d** (IC90 = 3.38 μM) superposed with the query (Hypo1); (**b**) Best aligned pose of compound **2g** (I C_90_ = 25.45 μM) fitted inadequately with the query (Hypo1); and (**c**) Best aligned pose of compound **1f** (I C_90_ = 26.84 μM) overlaid onto the pharmacophore model (Hypo1).

Figure 6a–c show the alignment of the hypothesis model with compounds **4d**, **2g**, and **1f** as representative examples. A closer look at the mapped structures revealed the importance of certain structural features for activity. Showing chemical features (3 HA, 1HD and 2HB) common to the variably active compounds, the quinoxaline-2-carboxamide-1,4-di-*N*-oxide scaffold is suggested to be essential for activity or may need further studies (Figure 6a). A slight displacement of the phenyl ring substituent on the amide moiety away from the plane of the aromatic pharmacophore center results in variation of activity (Figure 6b). At the C-7 of the quinoxaline ring, the displacement of the hydrophobic substituent away from the hydrophobic pharmacophore center (Figure 6b) or the presence of non-hydrophobic groups (Figure 6c) can partially explain their lack of activity. The rest of the features that are common for all compounds are the oxygen atoms adjacent to N1 and N4 positions of the quinoxaline scaffold, as hydrogen acceptor, the amide NH as hydrogen bond donor and the methyl or phenyl group at C-3 position.

### 2.2. Homology Modeling

**Figure 7:**
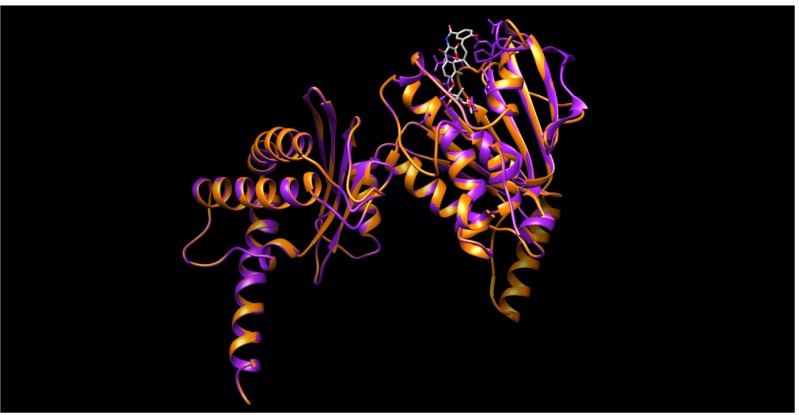
The structure of *M. tuberculosis* DNA gyrase subunit B (P0C5C5) (colored orange) is aligned with that of 1kijB.pdb (colored magenta) showing bound ligand, novobiocin, (colored grey).

Homology modeling and subsequently, a docking process were undertaken in order to inspect the prospective interactions between the quinoxaline 1,4-*N*-dioxide derivatives, and the active site of the *Mycobacterium* DNA gyrase B subunit. In the homology modeling study, the crystal structure of the gyrase B 43 K ATPase domain complex with the potent inhibitor novobiocin (1KijB.pdb) [22], was selected as the template structure. This particular template has been selected based not only on BLAST-p alignment but also on the structural similarity between our quinoxaline 1,4-di-*N*-oxide derivatives and co-crystallized novobiocin. During the homology modeling process the protein coordinates were first minimized using the AMBER94 force field, then the heavy atoms were modeled and followed by addition of all hydrogen atoms. The pair-wise percentage residue identity was determined as 41.321% between two chains, where the pair-wise RMSD values, for Cα atoms of the superimposed model and template, was 0.611 Å. In brief, the model structure comprises a compact single domain including an 8-stranded beta sheet and 6-alpha-helices and random coils (Figure 7).

### 2.3. Docking Procedure

**Figure 8:**
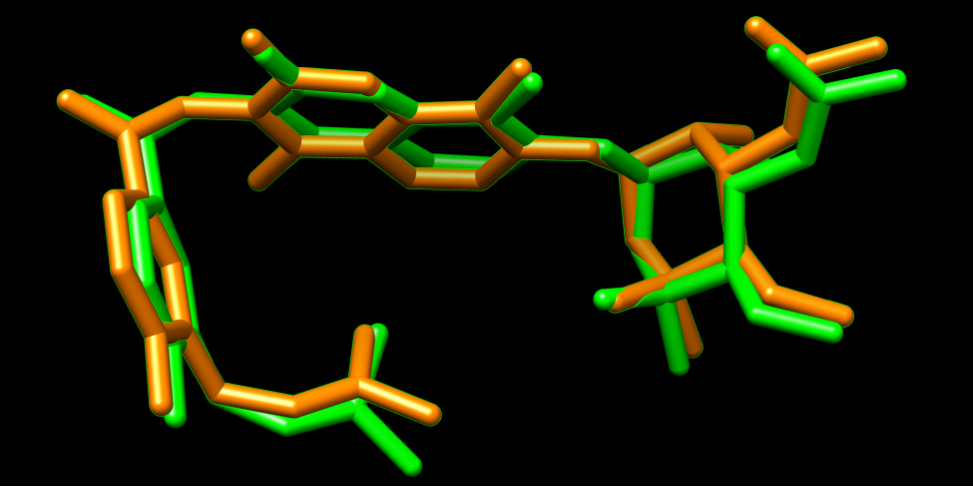
Superimposition of the co-crystallized novobiocin (from 1kijB.pdb, colored green) and the redocked.

**Figure 9:**
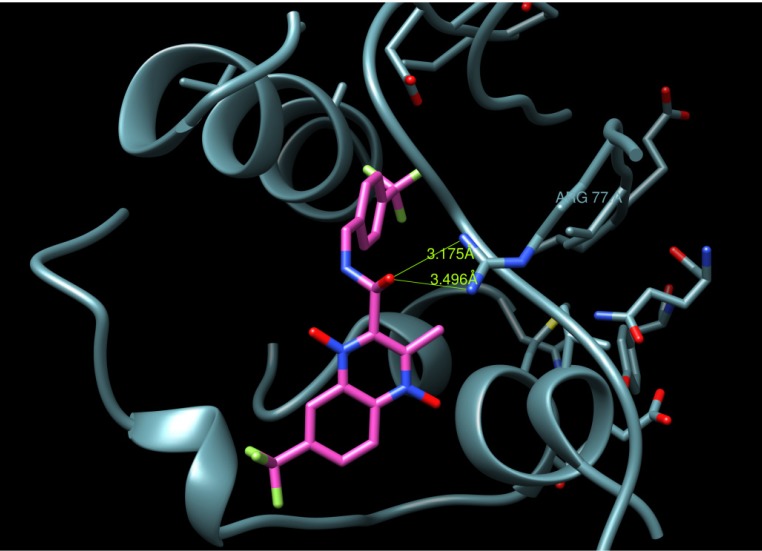
*M. tuberculosis* DNA gyrase subunit B (P0C5C5) homology modelled: the docked compound 4d (colored magenta), Hydrogen bond is displayed in green.

**Figure 10:**
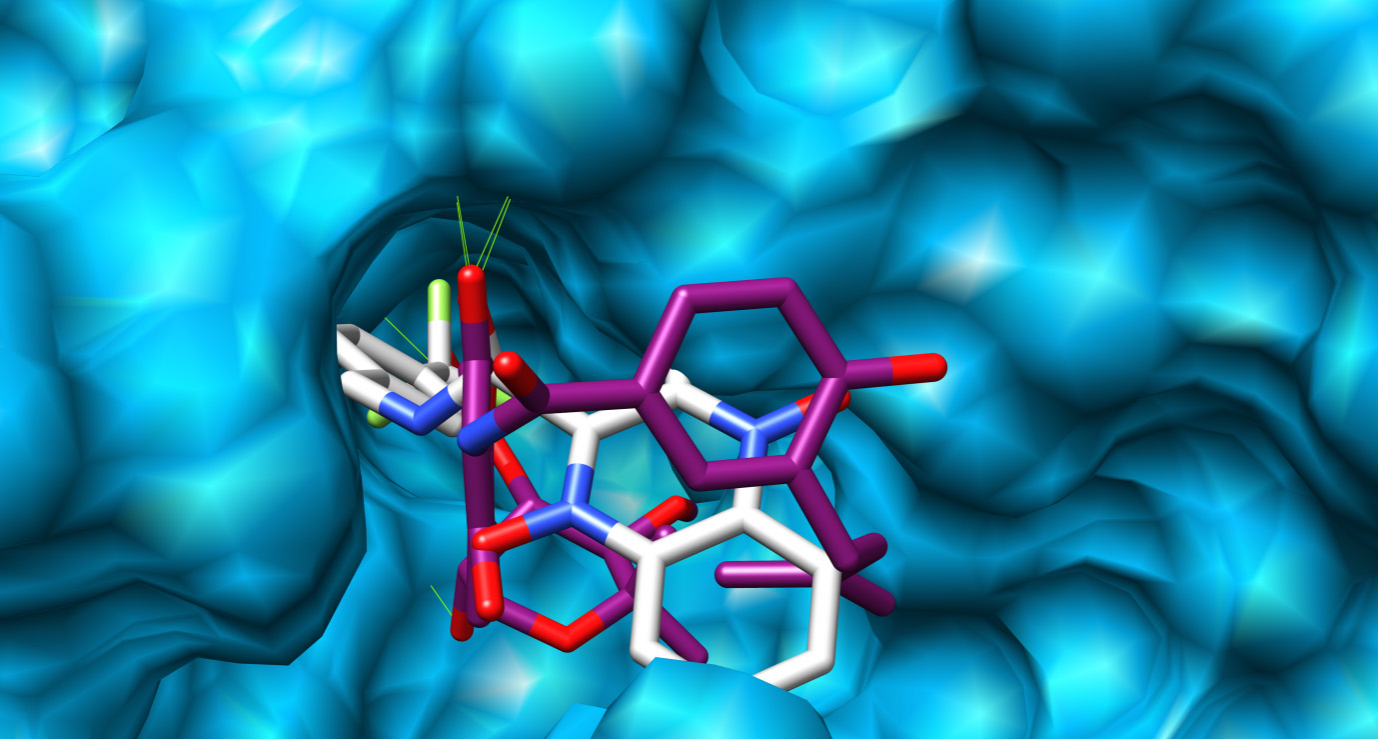
Binding site surface exploring compound **4d** (colored white) and novobiocin, X-ray-ligand (colored magenta).

**Figure 11:**
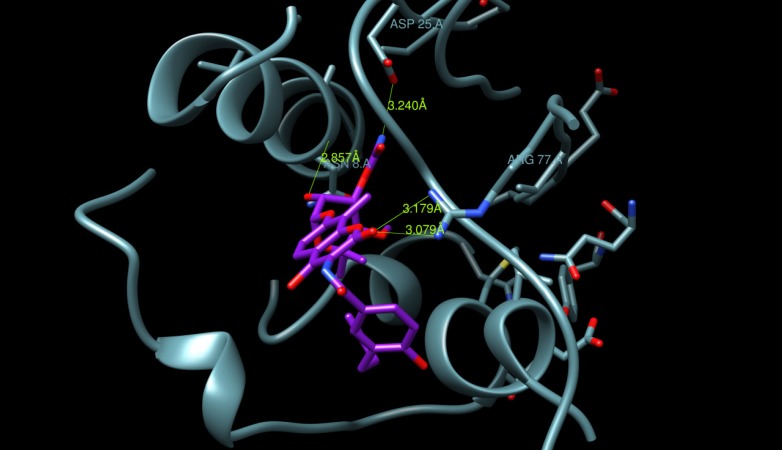
Binding site surface exploring novobiocin, X-ray-ligand (colored magenta).

A docking study was undertaken using Dock6.4 [23] in order to investigate the possible interactions between the designed compounds and the active site of the epidermal growth factor receptor (EGFR) and to compare it with the binding mode of the known 1kijB inhibitor, novobiocin. The X-ray structure of the enzyme bounded with novobiocin was taken from the protein data bank (PDB code: 1kijB) [22]. The RMSD value difference of 0.671415 Å of the pose of the non-restricted redocking of the X-ray structure of the gyrase inhibitor (novobiocin) from itself also confirmed the approach (Figure 8). The docking poses of compound **4d**, as an example of the designed compounds compared with that of the novobiocin, X-rary structure, are shown in Figure 9, Figure 10 and Figure 11, respectively. Occupying the same binding site as well as the coumarin moiety of the novobiocin X-ray structure, the quinoxaline-1,4-dioxide scaffold structure showed a different orientation. The substituted benzyl ring of the amide moiety is oriented in a binding pocket surrounded by ASN8, Ala9, Ala18, Gly27, ARG28, Gly29, Ile30, Gly83, Ser84 with hydrogen bonding between the oxygen atom of the amide group and the amino group of the side chain of Arg77.

Novobiocin structure (colored yellow).

## 3. Experimental

### 3.1. General

All molecular modeling studies were performed on PC Windows Vista Home Premium Intel(R) Core(TM)2 Duo, 1.83 GHz using the LigandScout program v3.1 1999–2013 (G. Wolber and Inte: Ligand GmbH, Vienna, Austria) [21] and the Dock6.4 program [23]. The quinoxaline 1,4-di-*N*-oxide derivatives used in this study are shown in Figure 1 and Figure 2. The biological data are cited from the literature [16].

### 3.2. Ligand Based Pharmacophore Modelling

The study was carried out using the software LigandScout (version 3.0). Using the default settings, the LigandScout program [21] was used to derive the 3D chemical feature-based pharmacophores from the structural data of the compounds **(1**, **2**, **4)** **a**–**g**, **3a**,**b** and **(5**–**9)** **a**, **b**, **e**, **g** [16] included in the modeling method. Prior to the generation of the pharmacophore hypotheses, the training set compounds **1b**,**c**,**f**, **2a**,**b**,**d**,**g**, **3a**, **4b**–**e**, **5a**,**e**, **6a**,**b**, **7b**,**e**,**g**, **9a**,**b**,**e** (Figure 1) were converted to 3D structures and used to generate diverse conformations. The diverse conformation generation protocol implemented in the LigandScout program was used to generate conformations using the best conformation model generation method. Other parameters like maximum number of 500 conformers, and an energy threshold value of 20 kcal/mol above the global energy minimum were chosen during conformation generation. During pharmacophore hypothesis generation four pharmacophoric features like hydrogen bond acceptor (HBA), hydrogen bond donor (HBD), ring aromatic (RA) and hydrophobic (HY) were selected based on the feature mapping results. All parameters were set to their default values.

#### 3.2.1. Pharmacophore Validation

The generated pharmacophore hypothesis was validated using leave-one-out and test set methods.

#### 3.2.2. Leave-One-Out Method

The pharmacophore hypothesis is cross validated by leave-one-out method. In this method, one compound is left in the generation of a new pharmacophore model and its affinity is predicted using that new model. The model building and estimation cycle were repeated until each compound was left out once [24]. This test was performed to verify whether the correlation coefficient of the training set compounds is strongly depend on one particular compound or not [24].

#### 3.2.3 Pharmacophore Validation using Test Set

Compounds **1a**,**d**,**e**,**g**, **2c**,**e**,**f**, **3b**, **4a**,**f**,**g**, **5b**,**g**, **6e**,**g**, **7a**, **8a**,**b**,**e**,**g**, **9g** (Figure 2) were selected as a test set. This method is used to elucidate whether the generated pharmacophore hypothesis is proficient at predicting the activities of compounds other than the training set and classifying them correctly in their activity scale. The conformation generation for the test set compounds was carried out in a similar way, like the training set compounds using BEST conformation analysis algorithm, implemented within the LigandScout program with setting values, as same as those used with the training set. The compounds associated with their conformations were subsequently carried out for pharmacophore mapping [24].

### 3.3. Homology Modeling

The building of the binding cavity in the specific and original size based on 1kijB (16-427) [22] was successfully obtained using BLAST alignment of the sequence of *M. tuberculosis* DNA gyrase subunit B (access number P0C5C5) [25] with the 1kijB.pdb sequence structure and followed by HH Search. Believed to be responsible for key interactions, the crystallized novobiocin structure and a water molecule were kept in their original positions for rebuilding during the modeling process. The BLAST module parameters were selected to run HHSEARCH of 50, minimal number of uncovered target residues to model an additional template of 25 and automated mode of SMR-pipeline. The obtained homology modeled-structure of *mtb*-DNA gyrase subunit B was used for the docking study in preparation of the input receptor files within Dock6.4 program.

## 4. Conclusions

In conclusion, a computational approach along with the 3D-QSAR and docking analysis was employed to identify molecular structural features required for effective antimycobacterial activity, with the aim of discovering drugs for treatment of *M. tuberculosis* infection. A reliable pharmacophore model was generated based on 22 training set compounds, which consists of one aromatic ring (A), four hydrophobes (HB), three hydrogen acceptors (HA) and one hydrogen donor (HD). This model revealed internal (R^2^ = 0.863) prediction of training set as well as external (Predr2 = 0.74) prediction of 21 compounds of test set. The quinoxaline-1,4-*N*-dioxide scaffold of the docked compound **4d** occupied the same binding site as the coumarin moiety in the novobiocin X-ray structure. Compound **4d** and the X-ray structure showed similar hydrogen bonding to the amino group of the side chain of Arg77. These findings could be exploited for future ligand design in order to obtain novel derivatives as inhibitors of *Mycobacterium tuberculosis*.
